# Improving healthcare for Aboriginal Australians through effective engagement between community and health services

**DOI:** 10.1186/s12913-016-1497-0

**Published:** 2016-07-07

**Authors:** Angela Durey, Suzanne McEvoy, Val Swift-Otero, Kate Taylor, Judith Katzenellenbogen, Dawn Bessarab

**Affiliations:** School of Dentistry, University of Western Australia, Perth, WA Australia; Centre for Aboriginal Studies, Curtin University, Perth, WA Australia; South Metropolitan Health Service, Fremantle, WA Australia; Faculty of Health Sciences, Curtin University, Perth, WA Australia; Western Australian Centre for Rural Health, University of Western Australia, Perth, WA Australia; Centre for Aboriginal Medical and Dental Health, University of Western Australia, Perth, WA Australia

**Keywords:** Aboriginal health, Community engagement, Partnership, Access

## Abstract

**Background:**

Effectively addressing health disparities between Aboriginal and non-Aboriginal Australians is long overdue. Health services engaging Aboriginal communities in designing and delivering healthcare is one way to tackle the issue. This paper presents findings from evaluating a unique strategy of community engagement between local Aboriginal people and health providers across five districts in Perth, Western Australia. Local Aboriginal community members formed District Aboriginal Health Action Groups (DAHAGs) to collaborate with health providers in designing culturally-responsive healthcare. The purpose of the strategy was to improve local health service delivery for Aboriginal Australians.

**Methods:**

The evaluation aimed to identify whether the Aboriginal community considered the community engagement strategy effective in identifying their health service needs, translating them to action by local health services and increasing their trust in these health services. Participants were recruited using purposive sampling. Qualitative data was collected from Aboriginal participants and health service providers using semi-structured interviews or yarning circles that were recorded, transcribed and independently analysed by two senior non-Aboriginal researchers. Responses were coded for key themes, further analysed for similarities and differences between districts and cross-checked by the senior lead Aboriginal researcher to avoid bias and establish reliability in interpreting the data. Three ethics committees approved conducting the evaluation.

**Results:**

Findings from 60 participants suggested the engagement process was effective: it was driven and owned by the Aboriginal community, captured a broad range of views and increased Aboriginal community participation in decisions about their healthcare. It built community capacity through regular community forums and established DAHAGs comprising local Aboriginal community members and health service representatives who met quarterly and were supported by the Aboriginal Health Team at the local Population Health Unit. Participants reported health services improved in community and hospital settings, leading to increased access and trust in local health services.

**Conclusion:**

The evaluation concluded that this process of actively engaging the Aboriginal community in decisions about their health care was a key element in improving local health services, increasing Aboriginal people’s trust and access to care.

**Electronic supplementary material:**

The online version of this article (doi:10.1186/s12913-016-1497-0) contains supplementary material, which is available to authorized users.

## Background

Different morbidity and mortality rates between Aboriginal and non-Aboriginal Australians have been well documented and persist despite dedicated government funding over the years to address the issue [1, 2]. In this paper, the term Aboriginal will be used to describe the local Indigenous population which is the preferred terminology used by the Western Australian (WA) Department of Health [3]. Life expectancy amongst Aboriginal Australians is around 10 years less compared to other Australians [4]. This disparity is the result of a range of complex causes, including the transgenerational negative effects of colonisation, dispossession and racism, and socioeconomic factors resulting from lower levels of education and employment, and higher rates of incarceration [5–9].

Aboriginal people can face many challenges when accessing mainstream services. These include unwelcoming hospital settings, lack of transport, mistrust of mainstream health care, a sense of alienation, and inflexible treatment options. This has resulted in an overall reluctance to attend services [10]. Research has also indicated that poor communication from health providers and lack of Aboriginal staff at health services exacerbates the problem [11, 12]. To resolve this, health services need to commit to developing respectful partnerships with local Aboriginal communities and increase the capacity of services to be more responsive to Aboriginal people’s requirements [13, 14].

In order to improve Aboriginal health and life expectancy, the Council of Australian Governments’ (COAG) agreed to the following funded National Partnership Agreements (NPA) [15] in 2008: 1) Closing the Gap (CtG) in Indigenous Health Outcomes and 2) Indigenous Early Childhood Development (IECD). A core function of the NPA was to build partnerships between government, service providers and local Aboriginal communities. The expected outcomes were to increase coordination between health service providers and to enhance health service access for Aboriginal people. A key element to achieve this is effective engagement with the Aboriginal community.

Community engagement has been variously described. Depending on the context it can mean consultation, communication, education, participation, partnership, collaboration and empowerment [16]. Involving the Aboriginal community in the decision making process requires partnership development and capacity building [17]. In a Queensland study [18], enablers and barriers to engaging Aboriginal people in a health promotion program were elucidated. Enabling factors included recognising the importance of local Aboriginal knowledge and cultural traditions, becoming familiar with the local Aboriginal community, and developing a local leadership network. These factors not only helped to build trust and gain acceptance but were necessary before implementing any interventions. The success of community engagement also relies on whether Aboriginal community members see benefits that outweigh the time–cost of participation [19]. Barriers included the negative impact of past interactions with health professionals, a narrow concept of health, and a lack of understanding of cultural differences [18].

Effective engagement with local Aboriginal communities as integral to improving Aboriginal health and increasing access to services was recognised by a metropolitan health service in the Department of Health, Western Australia. The Aboriginal Health Team (AHT) at the Population Health Unit (PHU) of the South Metropolitan Health Service (SMHS) in Perth had established and built strong relationships with local Aboriginal communities over recent years. The process was enhanced by the aforementioned Council of Australian Governments’ (COAG) funding for Aboriginal health. This funding was used to provide a range of local Aboriginal health initiatives, including the employment of Aboriginal health liaison officers at local hospitals (CtG), a community-based Aboriginal diabetes education and podiatry program (CtG) and an Aboriginal maternity program (IECD).

To ensure progress was being made, the SMHS was keen to evaluate the outcome of the community engagement process. Curtin University in Perth was awarded the contract to conduct the evaluation from a competitive tender process. This took place three years after the community engagement strategy was initially implemented.

This paper responds to calls in the literature [20, 21] to evaluate interventions for their effectiveness in improving healthcare for Aboriginal people and presents findings from the community engagement evaluation conducted by Curtin University [22] The objectives of the evaluation were to identify whether the community engagement process captured a range of community views on health service requirements and met Aboriginal participants’ expectations; whether those views were translated into actions by local health services, and whether Aboriginal people’s trust or confidence in health services changed as a result. The paper explains how the evaluation was conducted and provides evidence of critical success factors in engaging local Aboriginal communities and health providers. It also explores participants’ understanding of community engagement and offers suggestions for how community engagement can be sustained.

## Methods

### Setting

Perth is the fourth most populous city in Australia. This study pertains to the southern region of the city. The SMHS is divided into five health districts: Armadale, Bentley, Fremantle, Rockingham-Kwinana and Peel (Mandurah). Australian Bureau of Statistics (ABS) Census data show that in 2011, the resident population of SMHS was 867,371, of whom 15,317 were Aboriginal, accounting for 1.8 % of the population [23]. The Aboriginal population is dispersed across the five health districts, which has a total catchment area of almost 5000 km^2^.

### Initiating the community engagement process

Since 2008, the AHT at the SMHS PHU has actively engaged local Aboriginal people in improving their healthcare and driving the process of change. The AHT is made up predominantly of Aboriginal staff members with connections to the local Noongar Aboriginal community of the south-west of WA and other WA Aboriginal communities to the north. The staff bring many years of experience in health, including nursing, diabetes education and skills in chronic disease management and service delivery. In addition to cultural heritage, some staff members also have experience from a range of other sectors including housing, local government and child protection. This further enhances a holistic and social determinants approach to community engagement. The AHT bring an Aboriginal standpoint [24] to their lived experience and an understanding of Aboriginal ontology (ways of being), epistemology (ways of knowing) and axiology (ways of doing) in a contemporary Australian setting [25].

Prior to receiving COAG funding, community consultations were conducted to identify key health areas of concern for the Aboriginal community. Forums were held with up to 80 Aboriginal people attending area-wide gatherings. As part of the community, the AHT was well placed to engage with community members and elders, and was cognisant of the range of historical factors that led to Aboriginal people’s mistrust of mainstream health services. The staff acknowledged and respected the community’s apprehension and guarded replies to requests for further consultation about health care. Their approach to engaging the community focused on strengthening existing relationships and building the community’s trust through transparent communication. This involved sharing, reviewing and exchanging information with the community, and using community feedback to improve practice; an approach that continues today.

**Fig. 1:**
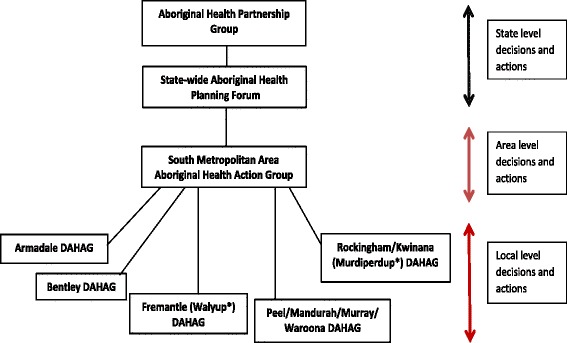
District Aboriginal Health Action Groups organised within the Western Australian Department of Health structure. ↔ Two way arrow shows the communication pathways. Community membership was essential at the local and area-wide levels. There was Aboriginal representation at each level, whether as community members or as Aboriginal staff members

In order to inform a SMHS Aboriginal Health Plan [26] and as a consequence of COAG funding being made available for Aboriginal health, five area-wide planning forums were undertaken between August and October 2009. The AHT coordinated and brought together local Aboriginal people, Aboriginal community-controlled health services, representatives from the Department of Health Western Australia, public hospitals, mental health, community health services in SMHS, and Divisions of General Practice. The aims were to identify areas of health need that could be funded through the COAG process; to inform ways to make health services more culturally secure and accessible; and to build relationships between service providers and the local Aboriginal community. An outcome of these forums that was was the establishment of five District Aboriginal Health Action Groups (DAHAGs) in 2010 that were based on *action* rather than simply *consultation,* and were located within the organisational structure of the Department of Health in Western Australia (see Fig. 1).

Each of the five south metropolitan districts formed a DAHAG. The DAHAGs met quarterly and comprised local community members (up to 10 per district), health service providers and an AHT representative from the PHU. Aboriginal community members were nominated by their peers and invited to sit on the DAHAGs for a two year term. The PHU arranged for governance training to be given on meeting procedures and chairing of meetings to community DAHAG members, in order to build their capacity. Community members were chosen as chairpersons. Each DAHAG had terms of reference, and agendas were set quarterly.

At the meetings, members discussed health issues with mainstream and Aboriginal-specific health service providers who reported on progress in improving healthcare. Beyond the meetings, health service providers were responsible for implementing recommendations from the DAHAGs to improve health service delivery for Aboriginal people. DAHAG community members were expected to disseminate information to their networks. Notably, community members were compensated for their time. An overarching Area Aboriginal Health Action Group (AAHAG) was established to oversee program implementation and health service changes. Two Aboriginal community members from each DAHAG were nominated to attend the AAHAG meetings.

Key objectives of the DAHAG process were to move beyond talk to action; avoid tokenism; identify local solutions to local Aboriginal health priorities; identify and action ways to deliver tailored Aboriginal health services; increase health service access for Aboriginal patients and improve coordination between local health services. DAHAGs were also involved in designing and monitoring local programs rolled out with the COAG CtG and IECD funding. Health service providers were tasked with committing to working in partnership with Aboriginal communities, to listen to their concerns and act on appropriate recommendations to effect change.

### Evaluation design

Qualitative methods used to gather data and gain insight into Aboriginal people’s experiences of the community engagement process are particularly useful when exploring the impact of social context in people’s lives [27]. In an Indigenous context, the evaluation team was particularly conscious of the work of Maori researcher, Linda Tuhiwai Smith [28] who has advocated for research with Indigenous people to include and value Indigenous knowledge and experience. Tuhiwai Smith argued that in the past, research has applied a white, colonial lens that has disregarded Indigenous experience, values, ways of knowing, and participation. To avoid replicating past research processes that have negatively impacted on Aboriginal people’s wellbeing, the evaluation team aimed to be collaborative, inclusive and respectful of Aboriginal ways. This approach takes into account differences in world views and the expectations that each party brings to the evaluation relationship [29]. It therefore becomes important in the evaluation process to ensure that there is ‘a shared understanding … between the evaluators and the Aboriginal participants about the aims and methods of the evaluation’ ([29], p.47).

The evaluation team was led by a senior Aboriginal researcher (DB) and five non-Aboriginal researchers. The team brought considerable experience in Aboriginal health and community research to the project. The Aboriginal project leader ensured the cultural integrity and safety of the evaluation by overseeing the data collection and analysis. This included regular ongoing discussions with the evaluation team and the AHT on recruiting participants, the evaluation design and interview questions, conducting interviews and organising the yarning circles, and thematic analysis and data interpretation guided by Aboriginal research methods [30]. The project leader also informed the community of progress which involved two team presentations that included disseminating findings and inviting feedback. These presentations were held at two AAHAG meetings attended by community DAHAG representatives from each district and health professionals. This inclusive and collaborative approach between the evaluation team, the AHT and the community ensured the research process was transparent and culturally appropriate.

Ethics approval for the evaluation was granted by the Western Australian Aboriginal Health Ethics Committee, the South Metropolitan Health Service Human Research Ethics Committee and the Human Research Ethics Committee at Curtin University.

### Recruiting participants

Participants were recruited using both purposive and selective sampling. In consultation with the AHT, prospective participants were identified and recommended through extensive community networks. The evaluation team contacted Aboriginal participants identified by the AHT by phone, email or by attending DAHAG meetings. Participants then identified other people to contact which led to a snowballing process of recruitment.

The evaluation team recruited participants in all five districts from the four stakeholder groups: 1) Aboriginal DAHAG members who accessed mainstream and Aboriginal health services but were not service providers; 2) Health Providers of Aboriginal Services (HPAS), including Aboriginal and non-Aboriginal professionals who catered mainly for Aboriginal people; 3) Aboriginal Specific Service Users (ASSU) who were local Aboriginal people but were not DAHAG members, and 4) Mainstream Health Service Providers (MHSP) who worked in services that engaged in the DAHAG process.

Evaluation team members attended DAHAG meetings in each district to recruit Aboriginal community members. At these meetings, the team introduced and explained the purpose of the evaluation and handed out an expression of interest form for members to complete with their contact details, which were collected at the conclusion of the meeting. The evaluation team followed up potential participants by phone or email to arrange an interview time.

Participants were provided with an information sheet that included the purpose of the evaluation, confidentiality assurances, the nature of their involvement and option to withdraw at any time without implications. All participants signed a consent form.

### Data collection

There were two main forms of data collection: one-on-one interviews and a culturally-appropriate form of group discussion known as a yarning circle. Yarning in research is defined as a conversation with a purpose and, in Aboriginal contexts, is similar to a semi-structured interview which applies narrative or storytelling in gathering information [30]. A recent publication has established yarning as a critical methodology in undertaking qualitative research with Indigenous communities that is culturally safe, yet maintains credibility and rigour [30]. Yarning in this evaluation was guided by the evaluation questions and began with ‘social yarning’ to develop rapport and a relationship with participants. This process then moved to exploring key issues related to the purpose of the research yarn.

Four key evaluation questions were developed in collaboration with the AHT who also had input into the methodology used by the evaluation team. The questions were:

1. Was the engagement process undertaken by the SMHS AHT sufficiently broad and participative to capture a range of community views?
2. Did the engagement process meet the Aboriginal participants’ expectations?
3. Were Aboriginal people’s views effectively translated into actions by health services?
4. What changes (if any) in trust/confidence in health services have been experienced at a personal, family or community level? [22]

(Additional file 1 provides supplementary questions that helped guide the evaluation).

### Data analysis

Interviews and yarning circles were recorded, transcribed verbatim and independently analysed and coded using NVivo 9.2 software (http://www.qsrinternational.com/) by two senior non-Aboriginal researchers. Thematic analysis was applied to code the data according to key themes which were further analysed for similarities and differences across the five districts. Applying triangulation across the four stakeholder groups, themes were cross-checked by the senior Aboriginal lead researcher to avoid bias and establish the reliability of the data interpretation.

Notably, participants were given the option to confirm the accuracy of the transcription prior to analysis. No participant was interviewed twice. Because the Aboriginal community in each of the five districts is relatively small, Aboriginal participants raised concerns about confidentiality, hence an important factor in this evaluation was protecting participant confidentiality. This was managed by aggregating data from interviews and yarning circles into specific groups and districts that were de-identified in quotes using acronyms and numbers for each group in each district; for example MHSP 5 is a mainstream health service provider from District 5 (see Table 1). Transcribed interviews were imported onto password protected computers and accessed only by the evaluation team.

**Table 1:** Participants’ de-identified information

Key stakeholder	Acronym	Districts	Total participants
District Aboriginal Health Action Group members	DAHAG	1–5	30
Aboriginal Specific Service Users	ASSU	1–5	12
Health Providers of Aboriginal Services	HPAS	1–5	4
Mainstream Health Service Providers	MHSP	1–5	14

Data were categorised and coded through several iterations into key themes and sub-themes, identifying similarities and differences within and between participant groups noting any emerging patterns. In the initial stages of coding, two members of the evaluation team coded three interviews, compared category choices and coding for similarities and differences that were then discussed and modified. In order to add rigour and reliability to the analysis, findings were further discussed with the Project Leader and modified till consensus was reached. This process continued with the remaining interviews. An evaluation rubric was developed so evaluative judgements could be made that supported robust data collection, analysis and reporting [31] by demonstrating the strength of responses to questions (Table 2). Rubrics are useful for assessing performance [32] and in this instance the evaluators were looking for evidence that showed how well the community engagement process performed in engaging with the Aboriginal community.

**Table 2:** Evaluating the strength of participants’ responses

Strength of response	Indicators
Very strong	Most participants referred to this theme, illustrating a majority experience
Strong	Many participants referred to this theme, illustrating a reasonably common experience/perspective
Mixed	Indicating diverse views in response to the question

## Results

Sixty participants across the five districts participated in one-on-one interviews and yarning circles which were conducted in 2012. This included 30 DAHAG members, one HPAS and 12 ASSU, all of whom were Aboriginal whilst the 14 MHSP comprised both Aboriginal and non-Aboriginal participants and three HPAS were non-Aboriginal. Thirty six people participated in the yarning circles and interviews were conducted with the remaining participants at a mutually convenient location (Table 3).

**Table 3:** Summary of yarning circles and interview participants according to district^a^

SMHS district	DAHAG members	HPAS	ASSU	MHSP	Total
1	6 (YC)	1	2	2	11
2	8 (YC) 2	0	0	7 (YC)	17
3	4 (YC) 2	1	4	1	12
4	3 (YC) 2	2	6 (YC)	1	14
5	2 (YC) 1	0	0	3	6
Total	30	4	12	14	60

*YC* Yarning circle

^a^Adapted from source [22]

Key themes indicated that participants in all groups (DAHAG, ASSU, HPAS and MHSP) felt that, despite challenges, the community engagement process was effective and working well (Tables 4 and 5). The community engagement process captured a range of views confirming that it was being driven and owned by the Aboriginal community whose needs were the focus of the process. Aboriginal people were included in decision making about their health care and, where possible, their views had influenced improvements to health services. Setting up the DAHAGs was a critical factor in the community engagement process.

**Table 4:** DAHAG and ASSU perspectives of the community engagement process

Was the engagement process sufficiently broad and participative to capture a range of community views?	Mixed evidence	Strong evidence	Very strong evidence
Community engagement process was effective			X
DAHAGs as effective strategy to recruit broad representation of community members		X	
Wide community representation	X		
Did the engagement process meet Aboriginal participants’ expectations?			
Aboriginal ownership and decision making, raising issues, advocating for change, supporting one another			X
Initial scepticism, apprehension about the initiative			X
Surprise that things were actually happening and that outcomes were emerging from the engagement process			X
Not tokenistic			X
Opportunity to break cycles and make change			X
Opportunity to meet with service providers and raise issues			X
Consultation has led to action, change and outcomes			X
Were those views effectively translated into actions by health services?			
Direct interaction with service providers			X
Service providers engaging with and listening to Aboriginal people			X
Accountability, commitment to improvement			X
Service providers reporting back and making changes to practice			X
Overall changes seen in health services in the region			X
Increased sensitivity of service providers to Aboriginal culture		X	
Increased flexibility in service delivery		X	
Community views are fed back with commitment to change at higher levels in health services	X		
Improved continuity of care, follow-up and referral of Aboriginal patients (Linkages created)		X	
Flexibility and availability of service provider		X	
Valuing and translating community input into services			X
Networking by service providers to raise awareness and build relationships to facilitate effective services		X	
What changes (if any) in trust/confidence in health services have been experienced at a personal, family or community level?			
Two-way capacity building (Aboriginal community and service providers)		X	
Consultation has led to action			X
Providing a service that is welcoming and respectful		X	
The community engagement process allayed concerns and built trust			X
Increase in health service use by Aboriginal people		X	
More health services are available for Aboriginal people			X
Aboriginal people having a voice in their health care			X
Aboriginal specific services			X
Relationship building between Aboriginal people and service providers			X
Culturally appropriate services			X
Relaxed, safe and welcoming atmosphere			X
Continuity of care		X

**Table 5:** MHSP and HPAS perspectives of the community engagement process

Mainstream Health Service Providers and Health Providers of Aboriginal Services	Mixed evidence	Strong evidence	Very strong evidence
Were Aboriginal community views effectively translated into action by health services?			
Direct interaction of communities with service provider			X
Respectful, accountable, committed			X
Consultation led to action and improved services			X
Inclusive of Aboriginal people			X
More equitable power balance		X	
Learning process		X	
Overall changes seen in health services in region			X
Increased sensitivity to Aboriginal culture and flexibility in service delivery			X
Building trust and providing a service that is welcoming and respectful			X
Community views are fed back and commitment to change at higher levels			X
Improved continuity of care, follow-up and referral of Aboriginal patients (Linkages created)		X	
Flexibility and availability of service provider		X	
Valuing and translating community input into services			X
Networking by service providers to raise awareness and build relationships to facilitate effective services			X
Increase in health service use by Aboriginal people		X	
More health services are available for Aboriginal people			X
New ways of working and delivering health services			X

Responses from DAHAG members and ASSU indicated very strong evidence that the community engagement process was effective, demonstrated by Aboriginal people being actively involved in decision making and advocating for change. Despite initial scepticism, there was strong evidence that engagement between DAHAG members and health providers on decisions about Aboriginal people’s health led to action and a commitment to improve health services, where possible. While responses were mixed about how representative of broad community views the engagement process was, the overall process nonetheless resulted in stronger relationships developing between community members and health services, improved health services that were more culturally appropriate, and increased access to and trust in services (Table 4).

Responses from MHSP and HPAS also indicated very strong evidence that the consultation process was effective in influencing service providers to listen, value and be more responsive to Aboriginal people’s concerns. This was demonstrated when health providers translated, where possible, community concerns into action by improving services so they were more flexible, welcoming and sensitive to Aboriginal culture (Table 5).

The concept of community engagement was explored further to elicit a deeper understanding of its meaning.

### Participants’ understanding of ‘community engagement’

Most DAHAG members discussed community engagement in interviews and yarning circles as bringing community together to talk about issues related to Aboriginal health and wellbeing:

*You have got everybody together and they are having their say and they know what is going on out there in their community, which before hasn’t been happening (DAHAG 1).*

The meaning of community engagement for health providers of Aboriginal health services (HPAS) was slightly different:

*… my understanding of community engagement is … instead of someone or a group of people, me included, sitting somewhere and … coming up with our plan of what the services might be or what they might look like, to actually go to the community and ask them and have them participate with you in those discussions, so that is my understanding of it. … That is the difference. … Before, I wouldn’t even have thought about doing that. It would have been … look at literature or look at other evidence and sort of plan without necessarily speaking to anyone directly that lives in whatever community we might be talking about. (HPAS 4)*

The above perspective of doing things differently was something that emerged from the DAHAG process and was a surprise for the participant who articulated this.

A mainstream health service provider added another perspective:

*Community engagement is having a two-way conversation that is respectful and that takes in the needs of the community and also best practices with the delivery … building trust … and making people feel welcome. (MHSP 3)*

The ‘two-way conversation’ took place at all DAHAG meetings. This respondent linked community engagement with best practice and the provision of a user-friendly and welcoming service for Aboriginal people.

### Regular meetings

The quarterly DAHAG meetings offer a point of connection between urban Aboriginal communities and local health services, resulting in greater community representation and input into local health issues. Periodic whole-of-area community forums were also held on a six-monthly basis to consult with the broader Aboriginal community and to present progress on the Aboriginal health initiatives that were implemented as a consequence of the COAG CtG and IECD funding.

*I think that this process that happened in South Metro was exceptional. No other region undertook community engagement to the extent or even in a whisper of what South Metro did to get an understanding of what the community’s needs are, what the community’s wants were, and engage with them at all … and the service providers within the area. (MHSP 5)*

The engagement process also enabled a coordinated response from the service providers in addressing Aboriginal health concerns at the local level:

*… the community consultations weren’t just about the community. So that was the beauty of these DAHAGs – they engaged both the service providers and the community and brought them into the room at the same time. (MHSP 5)*

In the eyes of the service providers, bringing them together with the community and encouraging dialogue has made this process unique. Meeting and talking with the Aboriginal community on a regular basis was not something that they were accustomed to, so this process enabled them to establish and develop relationships and hear firsthand some of the issues that prevented Aboriginal people from accessing services.

### Action group instead of planning or reference groups

The Aboriginal community wanted the name of the group to reflect its primary purpose of action and this was reflected in the DAHAG name:

*They didn’t want to be called planning forums because they were sick of planning. They wanted to call them action groups because it was signifying we were trying to do something rather than just do more planning. (MHSP 2)*

Many participants discussed the value of increasing the capacity of DAHAGs by encouraging members to choose their representatives, which placed authority in the hands of the Aboriginal community. Participants talked about how this process shifted the decision making about community health issues to Aboriginal people rather than a top-down approach with health providers identifying the health issues. This approach led to health service providers changing practice and that was viewed as positive at the community level.

*Aboriginal people say they are most consulted people in the world, so I think we can definitely say, ‘Yes, we have been consulted, but we have got an outcome from it and people will want to be consulted now because there has been action from it’. (HPAS 4)*

This comment reveals the participant’s perception that when action results from ‘talk’ and leads to changes that improve services, trust in the process of engagement is developed.

However, change was not universal; participants from one DAHAG group expressed frustration that service providers were not listening to them. The DAHAG process facilitated engagement and discussion between the community and health services. Key to the effectiveness of this process was health services being transparent about their limitations, such as the scarcity of resources to meet community expectations. Community expectations were managed through regular feedback and evidence of outcomes at each meeting.

### Aboriginal people making decisions about their healthcare

The structure of the DAHAG supported Aboriginal governance and leadership by ensuring that the chairperson was a community member with the meeting agenda and priorities set by the community, thereby helping to create a culturally safe space. This empowered and built the confidence and capacity of the community to speak up about their needs and concerns in relation to healthcare. Participants referred to DAHAGs being cultural governance committees where community was responsible for selecting who represented them on the DAHAGs:

*Aboriginal people have been the consultants. When they have allowed Aboriginal people to go to Aboriginal people and identify the support they need through the programs that health can give to them, I think that has been able to bring a sense of ownership, bring a sense of Aboriginal people caring for one another, Aboriginal people making decisions for one another. So … that has been great that Aboriginal people are doing it. (DAHAG 2)*

There was a sense that power relations had shifted. The Aboriginal community became an active decision maker and Aboriginal DAHAG members made up the majority on the committees where ‘*there is definitely a real sense that the power balance is much more evened out’ (MHSP 5).* Another unique aspect of the community engagement process was how the AHT approached the Aboriginal community and encouraged them to think about their health needs through community forums prior to the development of the SMHS Aboriginal Health Plan.

*The process was done a different way, instead of having the funding first and then just saying, ‘okay, this is the funding, these are our restrictions for the funding. How can we make it work for you?’ It was ‘if you had the funding what would you do with it? What is the best way? What programs do you want to see?’ Needs were identified and then we built it on from that way and I think that is where the success of the DAHAG came from. (HPAS 4)*

This more innovative and inclusive approach enabled service providers to hear and respond to what people said they needed. This resulted in new or modified services that were more appropriate and tailored to Aboriginal needs, for example, in maternity care.

*And maybe that is because they have not worked with Aboriginal people before, but I think (midwife) has been open to learning as well… it is a two-way street, I think, and I appreciate that. I appreciate not being treated like a child and not being assumed to be …on my second marriage because I had so many children and, you know, just the attitude that they get. It may not be overt but the insinuations are there and you pick up on that. And I know with [midwife], she follows through. You don’t see a different midwife every week or every time you go. (ASSU 1)*

### Accountability

Another unique aspect of the community engagement process was the establishment of accountability procedures, involving both community and health service providers.

*Two-way accountability, gaining respect from the community, keeping community engaged the whole time. Community were instrumental in how to go about the community engagement. (MHSP 2)*

Regular meetings facilitated continuity, follow up of issues and progress on actions.

*Looking at the engagement with the service providers and the DAHAG group, it has made it a lot more comfortable to speak up … Because I think ideally, we are sort of asked what we thought about certain things or how we would go about doing things … We help the different service providers, or give them ideas so that they can go away and look at some strategies. And with the service providers, they always come back to the DAHAG meetings, so you are getting updates, ‘Okay, this worked and that didn’t work’. (DAHAG 4)*

Accountability from health service providers was demonstrated when they reported back to the community on changes to health services following community recommendations made at DAHAG meetings, or on factors impeding change. DAHAG members were also accountable to the community they represented. This responsibility was twofold: not only to disseminate information on DAHAG business to the community, but also to feedback and discuss with health service providers any health concerns raised by the community.

*It is great to have community members ask questions about your service and it is good that they hold you accountable for what is happening, because without that process in place who knows how the money would be spent and whether the services would be done effectively. So [DAHAGs] are a governance not just for services, but for pretty much a lot of different planning and policies and services outside of health as well. (HPAS 4)*

Input from the community enabled service providers to tailor their services to meet Aboriginal people’s needs, therefore increasing the likelihood that their services would be used. Attending regular meetings and receiving feedback from the Aboriginal community about their services provided reassurance that they were delivering what people wanted or expected.

## Discussion

Findings suggest that overall, the community engagement process was broad enough to successfully capture a range of community perspectives and generally meet and sometimes exceed the expectations of those involved. Most participants agreed their views were heard and, when possible translated to action by health services, or, if not, reasons given about why this was not possible. Participants felt empowered to be actively involved in decisions about their health care, with training available in chairing and conducting DAHAG meetings with local health providers on how services could be designed and delivered to better meet Aboriginal people’s needs. Findings also showed that, as a result of this process, particularly with the establishment of the DAHAGs, participants’ confidence and trust were developing; this was seen as a welcome indicator that efforts were being made to improve services in light of Aboriginal people’s concerns.

The overall positive responses to the evaluation questions from the four stakeholder groups confirm the effectiveness of this community engagement approach. It is worth noting that most responses from all stakeholder groups indicated strong to very strong evidence of their approval of the community engagement process. However, responses in the ‘mixed’ category, while few, indicate room for improvement including strategies to facilitate a broader engagement with the community. Regular community forums provided a way to engage the broader Aboriginal community. DAHAG meetings were a successful way to build relationships between local Aboriginal people, the AHT and Aboriginal-specific and mainstream health services. The Aboriginal community and local health services were able to share knowledge and collaborate to improve health care and the experiences of Aboriginal people accessing these services.

Current literature suggests that engagement and partnership with Aboriginal communities is integral to any health policy or intervention to improve care [13, 14]. However, successfully building such partnerships requires a shift from expecting Aboriginal patients to adapt to the expectations of the health service to the health service being more inclusive, collaborative and flexible in responding to the needs of Aboriginal people in ways that are respectful and more likely to build trust and strengthen relationships.

Findings from the evaluation concur with literature which states that reciprocity or shared knowledge and understanding of Aboriginal health needs and recognising the importance of Aboriginal knowledge and culture can help establish and build intercultural partnerships [13, 14]. This evaluation supports other research revealing that as Aboriginal people become more involved in decision making about their health and healthcare, their confidence and sense of empowerment increase [33]. However, we suggest that empowerment requires a willingness to share and build on existing knowledge and skills. This two-way process involves Aboriginal communities learning about health services and the health system, and health services building their knowledge and skills about Aboriginal ways of working and Aboriginal culture.

A mutual commitment to establishing and maintaining the partnership is a key factor in its success. This includes agreeing on the purpose of the partnership and instituting effective processes around governance such as conducting meetings, designing work plans and documenting activities [34]. The DAHAGs were instrumental in establishing a governance structure to facilitate this process. The capacity of local Aboriginal communities was also developed with the assistance of the PHU in relation to the recruitment and training of community members.

From a service provider perspective, the innovative approach taken in this community engagement process shifted the power balance. From a top down model, where mainstream health services determined the care of Aboriginal people, to a grassroots model that encouraged the participation of the Aboriginal community. The DAHAGs became key players in identifying ways to improve the design and delivery of health services to better meet their needs. Health services that partner with an Aboriginal community to improve healthcare need to be transparent about what is possible in relation to health care. Failure to do this can compound past government and mainstream failures in addressing Aboriginal health, frustrating communities and sabotaging the community engagement process [19].

The majority of participants involved in the community engagement process commented on noticeable changes made by health service organisations in their area since it was initiated. Participants pointed out improvements in health service delivery at their local hospital and/or health agency. Most Aboriginal participants strongly supported the notion that consultation had led to action and that Aboriginal ownership was a feature of the DAHAG process.

Local Aboriginal health programs, established with the support of the DAHAGs, have shown demonstrable success. Two have been the subject of recent publications. The first program (Moorditj Djena) provides access to culturally secure diabetes education and podiatry care for Aboriginal people in the SMHS and has been well utilised by the local community [35]. The second program (Aboriginal Maternity Group Practice) provides antenatal care for Aboriginal women living in the SMHS area and has shown a considerable increase in the proportion of Aboriginal women choosing to birth locally and significantly improved neonatal outcomes [36]. The DAHAGs were instrumental in the design and roll out of these programs. Moreover, these programs, as well as others, have provided increased employment opportunities for local Aboriginal people.

The evaluation was limited by its focus on the small sample of participants. The evaluation team had asked participants if they could recommend other Aboriginal community members (non-DAHAG participants) or relevant stakeholders who had not participated in the community engagement process. The purpose was to gain as wide a range of perspectives as possible [37]. However, despite attempts of a team member to contact and follow up people by phone, recruiting Aboriginal people who had not participated in the community engagement process proved challenging. Thus, it remains unknown whether community members who chose not to participate in the community engagement process were unhappy with the process or did not engage for other reasons. However, given the number of Aboriginal people who accessed various services such as diabetes or maternity care, it appeared that awareness within the broader community was good. Aboriginal respondents speculated that information was disseminated via the ‘grapevine’ from DAHAG or community forum attendees. Hence, a non-participant would know who to ask for assistance about any health services queries.

The evaluation team identified key concerns that need to be addressed if the process of community engagement is to be sustained. First, ongoing funding needs to build on and strengthen existing partnerships. Second, regular reviews to ensure adequate Aboriginal representation across the region are warranted. Third, ongoing capacity building for DAHAG members through training, support and mentoring is needed to build knowledge, skills and experience, and increase employment opportunities.

Throughout the process the Curtin team was committed to working in partnership with the AHT to ensure that the evaluation process was respectful of the Aboriginal community. This was demonstrated by the team applying Indigenous methods to the evaluation process, providing regular feedback to the AHT, presenting findings to the community, and inviting feedback.

While the community engagement process may be adapted to other locations, findings may be different in other metropolitan, rural, remote or international settings. However, evidence from this evaluation suggests that application of the principles of this approach are worthy of consideration in the design and implementation of community engagement processes in other settings.

## Conclusion

The SMHS community engagement process demonstrated that actively engaging the Aboriginal community has been a key element in improving local health services for Aboriginal people. The formation of DAHAGs was a critical success factor in the engagement process and resulted in empowering the Aboriginal community to show leadership, drive the process, and communicate Aboriginal people’s concerns about healthcare to service providers. Action, rather than just planning, was a key function of the group. Health service providers worked with DAHAG community members to improve the cultural security of their services. The feedback loop that was established fostered a sense of accountability, transparency and trust and has resulted in better healthcare for the Aboriginal community.

## Abbreviations

AAHAG, Area Aboriginal Health Action Group; ABS, Australian Bureau of Statistics; AHT, Aboriginal Health Team; ASSU, Aboriginal Specific Service Users; COAG, Council of Australian Governments; CtG, Closing the Gap; DAHAG, District Aboriginal Health Action Group; HPAS, Health Providers of Aboriginal Services; IECD, Indigenous Early Childhood Development; MHSP, Mainstream Health Service Providers; NPA, National Partnership Agreement; PHU, Population Health Unit; SMHS, South Metropolitan Health Service; SMPHU, South Metropolitan Population Health Unit; WA, Western Australia.

## Additional file

Additional file 1: Improving healthcare for Aboriginal Australians through effective engagement between community and health services: guide to evaluation questions. (DOCX 23 kb)
