# Supplementary material for: Improving healthcare for Aboriginal Australians through effective engagement between community and health services
Source: BMC Health Serv Res. 2016 Jul 7;16:224. doi: 10.1186/s12913-016-1497-0 (PMC4936288; doi:10.1186/s12913-016-1497-0)
Supplement: Additional file 1: — Improving healthcare for Aboriginal Australians through effective engagement between community and health services: guide to evaluation questions. (DOCX 23 kb) [file 12913_2016_1497_MOESM1_ESM.docx]

| **Additional file 1: Improving healthcare for Aboriginal Australians through effective engagement between community and health services**  **Evaluation questions guide**  Key questions   - 1. Was the engagement process sufficiently broad and participative to capture a range of community views?   2. Did the engagement process meet the Aboriginal community’s expectations?   3. Were Aboriginal people’s views effectively translated into actions by health services?   4. What changes (if any) in trust/confidence in health services have been experienced at a personal, family or community level?   Supplementary questions  DAHAGs  What do you think ‘community engagement’ means?  How was the community involved in the development of the DAHAG model?  What do you understand is the purpose of the DAHAG?  What does ongoing attendance/participation in the DAHAG mean for you?  In what ways is the DAHAG process different from previous strategies to engage Aboriginal people?  Were you trained in how to run DAHAG meetings?  Do you think the engagement process allowed a representative view of all Aboriginal voices in the community?  How are the views of community members not part of DAHAG directly, involved in DAHAG considerations?  How effective are the DAHAG meetings from your experience?  What has changed as a result of the DAHAG process?  What has been the greatest outcome from this community engagement process?  HPAS  What do you think ‘community engagement’ means?  What does ongoing attendance in the DAHAG mean for you and your service?  What do you understand is the purpose of the DAHAG?  How are staff within your services selected to sit on the DAHAG?  How are staff within your service prepared and supported to attend DAHAG meetings?  How effective are the DAHAG meetings from your experience?  Do you think the engagement process allowed a representative view of all Aboriginal voices in the community?  Do you think the DAHAG process has changed the way mainstream providers deliver their services to the community?  Has your service/program changed as a result of ongoing engagement in the DAHAG process?  What has been the greatest outcome from this community engagement process?  MHSP  What do you think ‘community engagement’ means?  Please describe your involvement with the DAHAG process  What do you understand is the purpose of the DAHAG?  Do you think the DAHAG process differs from previous strategies to engage Aboriginal people?  How are staff within your services selected to sit on the DAHAG?  How are staff within your service prepared/supported to attend DAHAG meetings?  Do you think the engagement process has allowed a representative view of all Aboriginal voices in the community?  How effective are the DAHAG meetings from your experience?  In what ways has your service changed as a result of engagement in the DAHAG process?  What has been the biggest challenge for your service and/or staff in the DAHAG process? How can the process be improved to address this?  What has been the greatest outcome from this community engagement process?  ASSU  What do you think ‘community engagement’ means?  Do you know about the DAHAG process?  This service was put in place following community advice and direction as part of the DAHAG process. Have you been involved? Do you think this service is different to other services?  Do you feel comfortable using these services? Do you have any concerns about the services?  Do members of your family use this service?  Do you think this service is widely accepted in the Aboriginal community?  Do you think this service is suitable for Aboriginal people?  Do you think this service could be improved so more Aboriginal people use it?  Do you think you could approach the staff if you had any concerns about the service?  Do you go to any other services? Do you see similarities/differences between this one and others?  Do you know of other services that have been specially developed for Aboriginal people? |
| --- |
